# Evolution of crystal and electronic structures of magnesium dicarbide at high pressure

**DOI:** 10.1038/srep17815

**Published:** 2015-12-04

**Authors:** Dashuai Wang, Yan Yan, Dan Zhou, Yanhui Liu

**Affiliations:** 1 Department of Physics, College of Science, Yanbian University, Yanji 133002, China; 2 Applied Physics Institute, Changchun University, Changchun, 130022, China; 3 Laboratory of Clean Energy Technology, Changchun University of Science and Technology, Changchun, 130022, China; 4 Beijing Computational Science Research Center, Beijing 10000, China

## Abstract

Carbon-based compounds exhibit unexpected structures and electronic behavior at high pressure arising from various bonding features of carbon (e.g., *sp*, *sp*^2^ and *sp*^3^ C-C bonds). Here we report evolution of crystal structures of MgC_2_ in a wide pressure range of 0–200 GPa as predicted through *ab-initio* calculations in combination with an unbiased swarm structure search. Three pressure-induced structural transformations are unraveled, following the phase sequence of ambient-pressure *P*4_2_/*mnm* (α-phase) → *Cmcm* (β-phase) → *C*2*m* (γ-phase) → EuGe_2_-type *P-*3*m*1 (δ-phase), where significant C-C bonding modifications from C-C dimer to quasi 1-dimensionzigzag chain, to polymerized ribbon and then to winkled quasi 2- dimension graphite sheet are evident. The predicted β- and γ-phases with *sp*^2^ C-C hybridization are metals, while the δ- phase characterized by a *sp*^*3*^C-C hybridization is a narrow-gap semiconductor with a band gap of 0.667 eV. Strong electron-phonon couplings in the compressed β- and γ- phases arepredicted with β-phase showing a high superconducting critical temperature of 11.2 K. The current results indicate that pressure is effective in tuning the crystal and electronic structures of MgC_2_, which is expected to have impact on physical properties for potential applications.

Carbon has the ability to form various bonding states, e.g., graphite, diamond, nanotubes, fullerences, amorphous carbon and carbon-based compounds, *etc.*, which exhibit its unique physical and chemical property^1^,^2^,^3^,^4^,^5^,^6^,^7^. Carbides have been focus of intense for more than half a century and remain a major center of scientific of technology attention in advanced functional materials^8^,^9^,^10^. Among them, alkali and alkaline earth metal carbides have reignited great interest that show exceptional structural and electronic properties, as well as high-temperature superconductivity^11^,^12^,^13^,^14^,^15^,^16^,^17^.

MgC_2_ was originally prepared in 1910 with a unique *P*4_2_/*mnm* crystal symmetry, whereas the other dicarbides, namely, BeC_2_, CaC_2_, SrC_2_, and BaC_2_ adopt an *I*4*/mmm* crystal symmetry^12^,^13^. The crystal and electronic properties of the magnesium carbides have been systemically studied at ambient-pressure conditions^12^. Pressure induced reorientation of the dicarbide dumbbells similar to those in temperature-induced phase transitions has already been shown for LaC_2_ in its CaC_2_-type to ThC_2_-type phase change^18^,^19^,^20^. Recently, Li *et al.* reported that dumbbell carbon in CaC_2_ can be polymerized first into 1D chain and then into ribbon and further into 2D graphite sheet at higher pressure^20^. Especially, the high-pressure phase of CaC_2_ (*Immm*) with 2D graphite sheet has the high superconducting critical temperature Tc (7.9–9.8 K), which is comparable with the value of CaC_6_ (11.5 K)^21^. A pressing task is to understand the crystal and electronic structures of this special matter under the influence of external pressure conditions that may alter the underlying fundamental physics, which has motivated us to carry out the work.

Here, we unravel the convoluted structural evolution of MgC_2_ at high pressure using first-principles structural search method. We have identified three thermodynamically stable phases at high pressure. A systematic analysis of the electronic properties shows that the overlap between the conduction and the valence bands makes the β- and γ-phases metal, while the δ-phase is a narrow-gap semiconductor. Phonon-mediated superconducting behavior of two new metallic phases of MgC_2_ was revealed by exploring the electron–phonon coupling.

## Results and Discussions

### Crystal structures of MgC_2_

We performed variable-cell structure predictions with the simulation cell size of 1–4 formula at pressure of 0–200 GPa. At ambient pressure, our simulations revealed the experimental observed *P*4_2_/*mnm* phase (denoted as *α*-structure/phase) has a lower enthalpy than all other candidates, indicating that it is the thermodynamic ground state^12^. This proved the reliability of our method for application to this dicarbide system. Interestingly, with in the whole pressure range studied, three new low-enthalpy structures with space group of orthorhombic *Cmcm* (β- phase), monoclinic *C*2/*m* (γ-phase) and EuGe_2_-type hexagonal *P-*3*m*1 (δ-phase) were explored.

**Figure 1:**
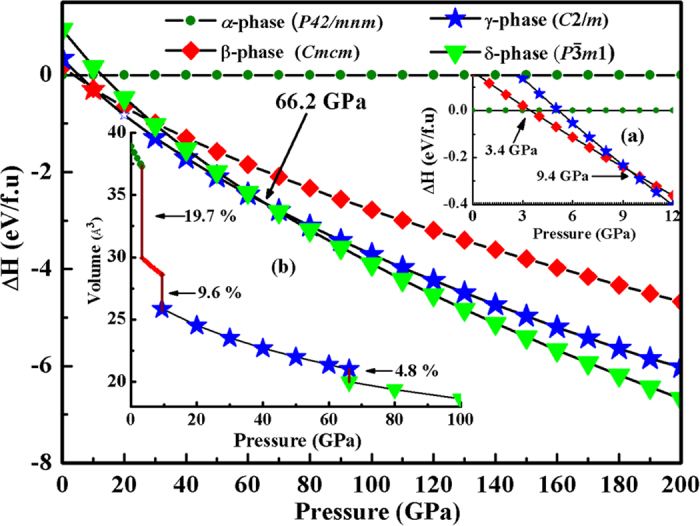
Enthalpies per formula unit of various structures as a function of pressure with respect to ambient pressure of the α-phase. Insert: (**a**) The enthalpies of the β- and γ-phases with respect to the *α*-phase. (**b**) The calculated equations of states for the predicted structures.

The enthalpies of these new structures with respected to the experimental structure are shown as a function of pressure in Fig. 1. In order to investigate the phase transition pressure clearly, the enthalpy differences vs. pressure for the β- and γ-phases are given in insert of Fig. 1(a). As can be seen that, for compressed MgC_2_, the ambient-pressure *α*-phase is the most stable structure below 3.4 GPa, and the β*-*phase become more favorable at the pressure range from 3.4 to 9.4 GPa, and then the *γ*-phase is stable from 9.4 to 66.2 GPa, above which *δ*-phase is the energetically much superior to the *γ*-phase from 66.2 to 200 GPa. The current experimental techniques are readily accessible to these extreme conditions. The pressure evolution of the unite cell volume of MgC_2_ in the α-, β-, γ- and δ-structures are depicted in insert of Fig. 1(b). The abrupt volumecollapses of about 19.7%, 9.6% and 4.8% around 3.4, 9.4 and 66.2 GPa, respectively, indicating the first-order nature of these phase transitions in MgC_2_. No imaginary frequencies are observed throughout the whole Brillouin zone, declaring that the three novel phases are dynamically stable at studied pressure region.

**Figure 2:**
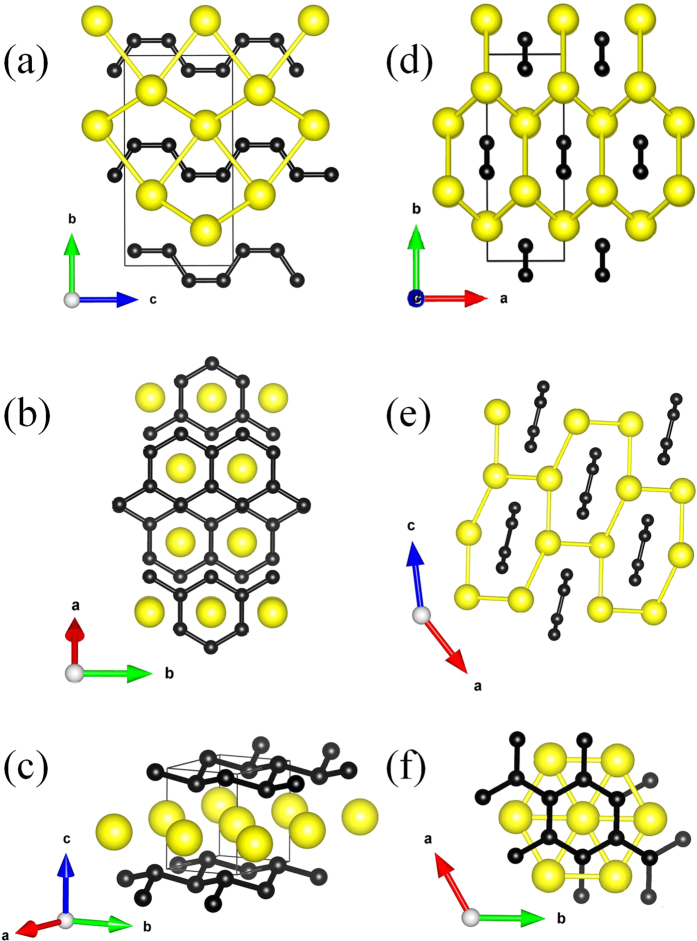
Crystal structures of for MgC_2_. The yellow and black balls represent Mg and C atoms, respectively. (**a,d**) The β-phase. (**b,e**) The γ-phase. (**c,f**) The δ*-*phase.

**Figure 3:**
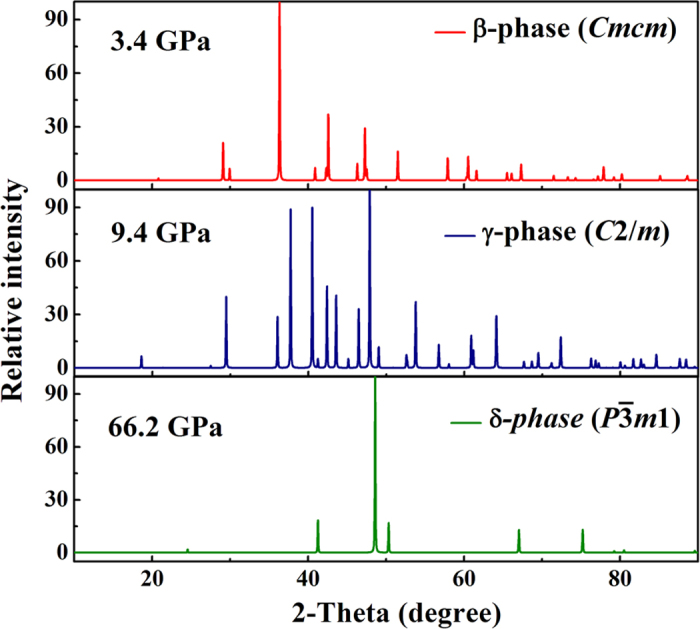
Simulated X-ray diffraction of MgC_2_. Thecalculated power X-ray diffraction (λ = 1.541 Å) for β-phase at 3.4 GPa (**a**), γ-phase at 9.4 GPa (**b**), and δ-phase at 66.2 GPa (**c**).

The optimized structure parameters at related pressures are listed in Table 1. The atomic arrangements of competing structures are shown in Fig. 2. The calculated lattice constants of the ambient-pressure have been widely demonstrated, consistent results with power X-ray and neutron powder diffraction measurements^12^. For the β*-*phase, the equilibrium lattices are *a* = 3.183 Å, *b* = 8.536 Å and *c* = 4.410 Å at 3.7 GPa. Four Mg atoms occupy the wyckoff 4*c* site and eight C atoms lie in 8*f* site in the unit cell. The β-structure is similar to the recently predicted of CaC_2_ for *Cmcm* phase^20^. Figure 3 shows the simulated X-ray diffraction data of these structures, indicating that their structures are differenr from each other.

In this structure, one-dimension carbon chain along *z* direction lies in the center of the structure, in which the Mg atoms construct cylinder with hexagon cross-section. With increasing pressure, the γ-structure contains four molecules per unite cell. The optimized structural parameters are *a* = 8.321 Å, *b* = 2.609 Å and *c* = 9.304 Å. Four Mg atoms hold the wyckoff 4*i* site and the in-equivalent of two C atoms also lie in *4i* site in the unit cell, respectively. The results show that carbon atoms are polymerized into ribbon with a six-membered ring. A carbon quasi 1-dimension ribbon lies in the center of cylinder constructed by Mg atoms. For the δ-phase with a EuGe_2_-type structure, including one molecule in unite cell, the equilibrium lattice are *a* = *b* = 2.525 Å and *c* = 3.623 Å at 66.2 GPa. One Mg atom occupies the wyckoff 1*b* site and two C atoms at 2*d* site. The plans of Mg atoms separate the hexagonal honeycombed layers of carbon atoms. It is obviously that the δ-phase consists of the bulking honeycomb-layered of carbon atoms separate by planes of Mg atoms.

**Figure 4:**
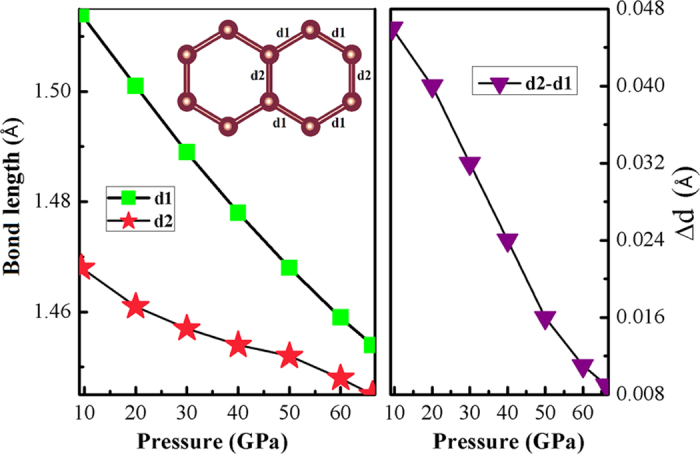
C-C bond lengths in the γ-structure as a function of the pressure. Left: the two kinds of C-C bonds length in the γ-phase as a function of the pressure. Right: the difference (∆*d*) between *d*_1_ and *d*_2_ as a function of pressure.

We investigate the nature of carbon-carbon bonding in MgC_2_ with increasing pressure. The carbon-carbon bonding behavior reveals significant change with the external pressure and chemical precompression. At ambient pressure, the length of isolated dumbbell C-C bond in the α-phase (*P*4_2_/*mnm*) is 1.253 Å, which is nearly identical to the experimental value of 1.215 Å^12^. With increasing pressure, the distance between the isolated dumbbell decreases, resulting in carbon atomic chain formed in the β-phase (*Cmcm*), consistent with the increase in the C-C distance (1.458 Å and 1.401 Å). Under further compressed, the carbon atomic chains polymerize to a well-organized arrangement, transforming to the monoclinic (*C/*2*m*) γ-phase. It is obviously that the carbon nanoribbons appear between neighboring Mg atomic layers. The two types of in-equivalent C-C bond lengths in the γ-structure referred as *d*_1_ and *d*_2_ as a function of the pressure is plotted as a function of pressure in Fig. 4. The difference (*∆d*) between *d*_1_ and *d*_2_ in ribbon with six-membered carbon ring is also explored. At higher pressure, the difference *∆d* begins to decrease more smoothly and reaches 0.009 Å at 66.2 GPa. The C-C bond lengths are 1.445 Å and 1.454 Å close to that of graphite (1.420 Å). However, the C-C bond lengths in honeycomb layer is 1.544 Å in the δ-phase, larger than those of the γ- structure at 66.2 GPa. The increased bond length induced the well organized carbon six-ring collapse into the winkled quasi 2-dimension graphite sheet. Due to the occurrence of graphite sheet between neighboring Mg atomic layer, the γ-phase can be regarded as one of folded graphite intercalation compounds.

### Electronic structuresof MgC_2_

**Figure 5:**
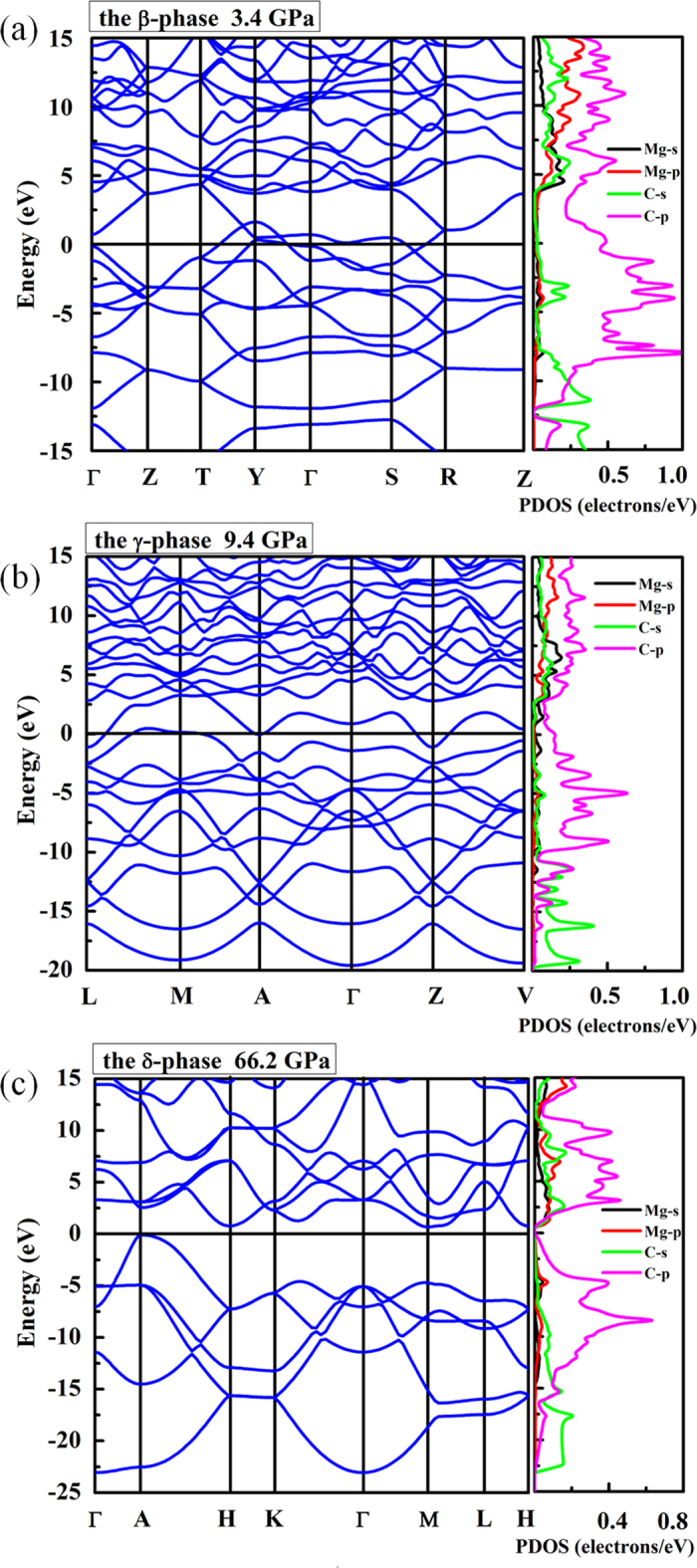
The electronic properties of MgC_2_. Band structure and electronic density of states of MgC_2_ for the (**a**) β-phase, (**b**) γ-phase, (**c**) and δ-phase. The zero of energy is at the Fermi level.

**Figure 6:**
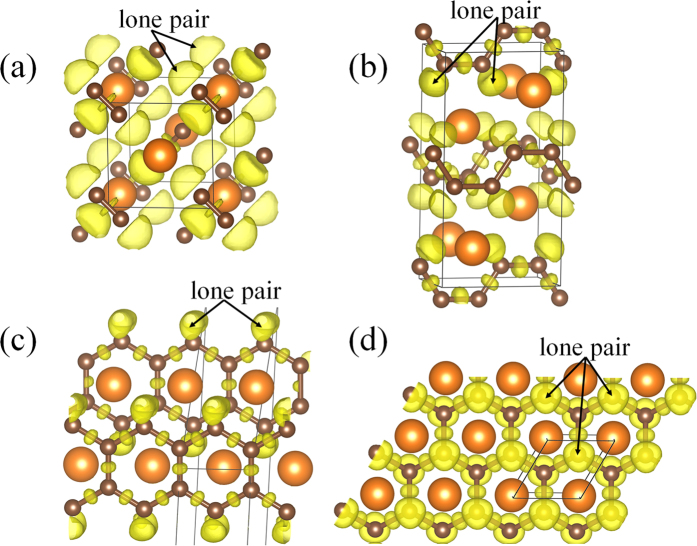
Calculated ELF of MgC_2_. Contours of the ELF of MgC_2_ for the (**a**) α-phase, (**b**) β-phase, (**c**) γ-phase and (**d**) δ-phase with isosurface of 0.86.

The calculated band structure and projected density of states are shown in Fig. 5. The ambient-pressure α-phase is an insulator with a larger indirect band gap of ~2.643 eV. The predicted overlap between the conduction and the valence bands shows the β- and γ-phases with *sp*^2^ hybridization are metallic with C-*p* orbital dominating the density of states at the Fermi level. However, there are few C-*s* electrons and Mg-*s, p* electrons near the Fermi level, showing a pressure-induced charge transfer from C-*s* and Mg-*s*, *p* to C-*p* electron. In the α-phase, the value of the band gap decreases with the increasing pressures as the molar volume decreases along with the *van der* Walls gap reduces, the orbital overlaps across the gap are enhanced and this would reduce the energy separation between bonding and anti-bonding interactions. The reduction of the band gap is a promising signal for reaching a metallic state. At 66.2 GPa, the δ-phase characterized by *sp*^3^ hybridization is most thermodynamically stable and is semiconductor with an indirect band gap of 0.667 eV. The significant evolution of electronic structures from *sp*^2^ to *sp*^3^ hybridization of carbon atoms between the γ- and δ- phases is similar to the fact of graphite transforms to diamond under sufficient compression. The pressure-induced insulator-metallization-semiconductor and related propertiesincent us to calculate the electron localization behavior of MgC_2_. Therefore, we have performed a chemical bonding analysis *via* calculation of the electron localization function (ELF)^22^. The calculated ELF isosurface (0.86) for the α-structure shows that two C atoms bond together to form a double bonded C_2_ dimer with a stable and localized lone-pair nonbonding state, as shown in Fig. 6. With increasing pressure, the distance between the isolated dumbbell decreases, constructing carbon atomic chain with armchair-type carbon chains in the β-phase. One can see that each C has one lone pair of electrons. In the γ-structure, the ribbon with a six-member carbon ring has the feature of *sp*^2^- like nature with each inner C atoms forming three C-C covalent bonds, while each outer C atoms has one electron lone pair and two C-C covalent bonds, while the remaining 2*p*_*z*_ electrons of the C_6_ ring form a delocalized *π* system. In the δ-phase, the winkled honeycomb-layered structure lacks the system of delocalized *π* bonds with mobile electrons and has more localized electrons, as a result of which the obviously *sp*^3^ hybridization constructed. Each C atom has one electron lone pair and three covalent C-C bonds resembling diamond structure, accompanied with to appear insulator.

### Electron–phonon coupling of MgC_2_

**Figure 7:**
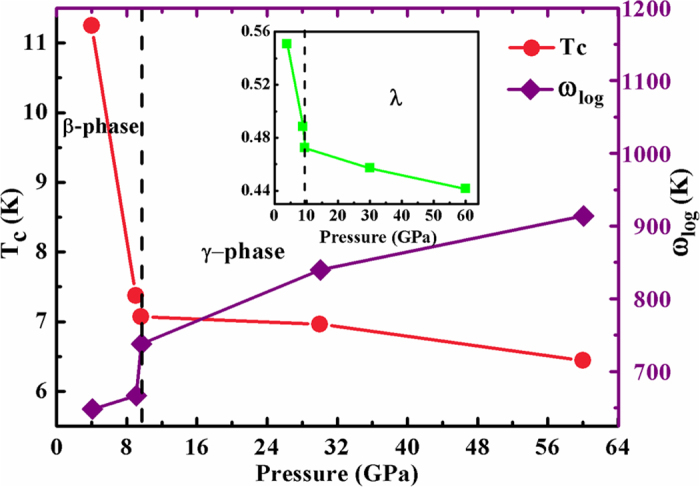
Electron-phonon coupling of MgC_2_. Calculated Tc values and the logarithmicaverage of the phonon frequency of ω_log_ as a function of pressure. Inset shows the electron-phonon coupling integration of λ(ω) as a function of pressure.

The high-pressure β- and γ-phases of MgC_2_ exhibit charming features in the electronic band structure. As shown in Fig. 5, the electronic bands of the β-phase crossing the Fermi level along the Y-Γ-S-R are quite flat, and a narrow energy window located at the Fermi level results in large electronic density of states near the Fermi energy. The corresponding restricted conduction electrons near the band gap possess large effective mass with their group velocities approaching zero. However, the bands along the Γ-Z-T direction steeply cross the Fermi level, providing itinerant electrons with high conduction electron velocity. Above all, such flat bands with highly mobile and localized electrons should provide the strong electron-phonon coupling necessary for superconductivity. This encourages us to further investigate the superconductivity of MgC_2_ at high pressure. The calculated Tc values and logarithmicaverage frequency ω_log_ and the electron–phonon coupling parameter λ of MgC_2_ at different pressure were plotted in Fig. 7. For the β-phase, at 4 GPa, the coupling parameter λ is 0.558 with the logarithmicaverage of the phonon frequency ω_log_ of 648 K. Using the strong coupling Allen-Dynes equation and an extension of the McMillian theory^23^,^24^, with a nominal Coulomb pseudopotential parameter (μ^*^) of 0.1 the estimated superconducting critical Tc is 11.3 K. With the increase of pressure, the obtained *T*_*c*_ become slightly lower as 7.4 K at 9 GPa with the slightly smaller of λ 0.488 than that of values at 4 GPa. The calculated results show that ω_log_ has positive pressure dependence, and coupling parameter λ decreases with pressure. For the γ-phase, the estimated superconducting critical *T*_*c*_ is 7.1 K at 9.6 GPa, which is comparable with that of β-phase due to the similar of electron-phonon coupling integral (λ) 0.472.

## Conclusion

In summary, we have reported three high-pressure phases of MgC_2_, *Cmcm* (β-phase), *C2m* (γ-phase), *P-*3*m*1(δ-phase), which are stable in the pressure ranges of 3.4–9.4 GPa, 9.4–66.2 GPa and 66.2–200 GPa, respectively. The structural, dynamical, and electronic properties of high-pressure MgC_2_ were systemically investigated up to 200 GPa. The predicted α- to β-phase structural transition is accompanied with a metallization by band gap closure. The γ-phase transfers to the δ-phase with a narrow band gap characterized by C-C *sp*^3^ hybridized. Strong electron-phonon coupling in the compressed β- and γ-phases is found, in particular for the β-phase with carbon sp^2^-hybridization, which has the highest λ 0.558 value, leading to its high superconducting critical temperature Tc of 11.3 K, which is comparable with the 11.5 K value of CaC_6_. These results show that pressure has a strong influence on the fundamental crystal and electronic structure of MgC_2_, and as a result, the sensitive pressure tuning of the electronic properties offers an effective tool to modulate a wide range of physical properties for its potential applications.

## Methods

The search for the stable structures is based on the CALYPSO (Crystal structure Analysis by Particle Swarm Optimization) methodology^25^,^26^,^27^ and the first-principles calculations using a global minimization of free energy surfaces. The remarkable feature of this methodology is the ability of predicting the stable structure with the knowledge of chemical composition under given external pressures^28^,^29^. Enthalpy calculations and geometry optimizations were performed within the framework of the Perdew-Burke-Ernzerhof parametrization for the exchange-correlation functional as implemented in the VASP (Vienna *ab initio* simulation package) code^30^. The projector augmented wave method^31^,^32^ has been adopted, with 2*p*^6^ 3*s*^2^ and 2*s*^2^ 2*p*^2^ treated as valence electrons for Mg and C atoms, respectively. For Brillouin zone integration, we used the Monkhorst−Pack scheme^33^ and checked convergence of ground state calculations with uniformly increasing *k*-point meshes for each structure. We used cutoff energy of 900 eV for the expansion of the wave function into the plane-wave basis-set. Monkhorst-Pack *k*-pointmeshes with a grid of 0.03 Å^−1^ for Brillouin zonesampling were chosen to achieve the total energy convergence of less than 1meV/atom. The phonon calculations were carried out by using a supercell approach as implemented in the PHONOPY code^34^. Electron–phonon coupling calculations were performed using the planewave pseudopotential method and density-functional perturbation theory as implemented in the QUANTUM ESPRESSO with a kinetic energy cutoff of 70 Ry^35^. 4 × 4 × 3 and 4 × 4 × 2 *q*-meshes in the first Brillouin zones were used in the EPC calculations for the β- and γ- structures, respectively.

## Additional Information

**How to cite this article**: Wang, D.-S. *et al.* Evolution of crystal and electronic structures of magnesium dicarbide at high pressure. *Sci. Rep.* **5**, 17815; doi: 10.1038/srep17815 (2015).

## Figures and Tables

**Table 1:** Lattices Parameters and atomic coordinates of α-, β-, γ-, and δ- phase at 0, 3.4, 9.4, and 66.2 GPa,respectively.

	Lattice Parameters (Å)	Atoms	x	y	z
α-phase	*a* = 3.944 (3.934^a^)	Mg(2*b*)	0.0	0.0	0.5
0 GPa	*b* = 3.944 (3.934^a^)	C(4*f*)	0.1123	0.1123	0.0
	*c* = 5.008 (5.021^a^)				
β-phase	*a* = 3.183	Mg(4*c*)	0.0	0.1658	0.250
3.4 GPa	*b* = 8.536	C(8*f*)	0.000	0.4286	0.0911
	*c* = 4.410				
γ-phase	*a* = 8.321	Mg(4*i*)	0.0549	0.0	0.7096
9.4 GPa	*b* = 2.609	C1(4*i*)	0.6145	0.0	0.2407
	*c* = 9.876	C2 (4*i*)	0.0626	0.0	0.1217
δ-phase	*a* = 2.525	Mg(1*b*)	0.0	0.0	0.500
66.2 GPa	*b* = 2.525	C(2*d*)	0.6667	0.3333	0.0718
	*c* = 3.623				
